# Effects of an active ankle exoskeleton on the walking biomechanics of healthy men

**DOI:** 10.3389/fbioe.2025.1533001

**Published:** 2025-03-14

**Authors:** Sridevi Nagaraja, Jose E. Rubio, Junfei Tong, Aravind Sundaramurthy, Anup Pant, Meredith K. Owen, Michael A. Samaan, Brian Noehren, Jaques Reifman

**Affiliations:** ^1^ Department of Defense Biotechnology High Performance Computing Software Applications Institute, Telemedicine and Advanced Technology Research Center, Defense Health Agency Research and Development, Medical Research and Development Command, Fort Detrick, MD, United States; ^2^ The Henry M. Jackson Foundation for the Advancement of Military Medicine, Inc., Bethesda, MD, United States; ^3^ Department of Physical Therapy, University of Kentucky, Lexington, KY, United States; ^4^ Department of Kinesiology and Health Promotion, University of Kentucky, Lexington, KY, United States

**Keywords:** exoskeleton, individualized models, walking, load carriage, musculoskeletal biomechanics

## Abstract

Active lower-body exoskeleton devices can decrease the energy requirement of the human body by providing mechanical assistance to lower-body muscles. However, they also alter gait kinematics and kinetics, and it is not well understood whether such alterations are detrimental or beneficial to the human body. In this pilot study, we investigated the impact of walking with an ankle exoskeleton device on the biomechanics of men while carrying a heavy load. We collected computed tomography images and motion-capture data for five young, healthy men who walked 5 km (∼60 min) with a 22.7-kg load, with and without an active ankle exoskeleton (the ExoBoot EB60). We developed personalized musculoskeletal models and calculated the joint kinematics and kinetics for each participant under each walking condition. Without the ExoBoot, at 5 km compared to 0 km, on average, the peak trunk flexion angle increased by ∼35% and the stride length increased by ∼3.5%. In contrast, with the ExoBoot, the magnitude of the corresponding increases was smaller (∼16% and ∼2%, respectively). After the 5-km walk, compared to walking without the ExoBoot, its use considerably altered hip-related biomechanical parameters, e.g., it increased hip abduction angle by ∼17%, increased hip flexion moment by ∼3.5%, and decreased hip adduction moment by ∼19%. Finally, irrespective of distance, ExoBoot use significantly increased the stance duration and peak ankle plantarflexion angle (*p* < 0.001). Overall, the use of the ExoBoot induced beneficial alterations in stride length and trunk-, ankle-, and hip-related parameters for men walking with load carriage. The quantitative analysis provided by this pilot study should help guide future investigations and inform the development of standards for safe and effective use of emerging exoskeleton technologies.

## 1 Introduction

Military operations often require Service members to carry heavy loads, which in the U.S. Army typically range from 22.7 to 31.7 kg (Knapik et al., 2012). Such loads have been associated with musculoskeletal injuries of the lower extremities and lower back, affecting Service members’ health and reducing Force Readiness and Lethality (Knapik et al., 2012; Orr et al., 2015; Orr et al., 2017). In fact, musculoskeletal injuries account for more than 2 million medical visits annually, imposing a considerable cost on the military healthcare system (Department of the Army, 2011). Thus, the U.S. Army is continually exploring ways to reduce the incidence of such injuries. For instance, previous studies have found that during strenuous physical activities, such as walking and running with or without load carriage, reducing the stride length decreases the load to the bones, offering an opportunity to help reduce stress-fracture risk to the tibia for both men and women (Willy et al., 2016; Sundaramurthy et al., 2023). As an alternative to modifying the stride length, which requires a conscious effort by the individual, wearable exoskeleton devices offer the potential to enhance an individual’s strength and endurance (Bogue, 2015), allowing Service members to carry heavier loads for longer distances.

Lower-body exoskeleton devices offer a particularly promising solution because they increase mobility, physical endurance, and load-carrying capacity while counteracting overstress on the lower back and legs, allowing Service members to carry a 90.7-kg load while marching at a speed of 4 km/h for up to 20 km (Bogue, 2015). Exoskeleton devices are typically classified as passive or active based on their mechanism of action. Passive exoskeleton devices usually leverage their mechanical design to store and release energy without powered components, however, their ability to control the amount of the enhanced mechanical power is often limited (Lee et al., 2020; Johnson et al., 2023). In contrast, active exoskeleton devices assist the wearer through powered actuation via electronic control systems and offer a higher degree of mechanical-power modulation under different conditions. Indeed, many emerging active lower-limb exoskeleton devices apply torque at either the ankle joint (Sawicki and Ferris, 2008; Malcolm et al., 2013; Mooney et al., 2014a; 2014b; Jackson and Collins, 2015; Mooney and Herr, 2016; Zhang et al., 2017) or the hip joint (Ding et al., 2017; Panizzolo et al., 2019), and operate in tandem with the wearer to augment their walking capacity while carrying heavy loads. Active exoskeleton devices have demonstrated favorable results, including reductions in metabolic energy requirements by up to 14% (Gordon and Ferris, 2007; Sawicki and Ferris, 2009a; Mooney et al., 2014b; Koller et al., 2015; Ding et al., 2017; Zhang et al., 2017; Panizzolo et al., 2019; Sawicki et al., 2020) and reductions in the activation of different muscle groups, such as the soleus and quadriceps, by ∼35% (Gordon and Ferris, 2007; Zhu et al., 2021).

Nevertheless, there is conflicting evidence in the literature as to whether the biomechanical changes induced by exoskeleton devices are beneficial or detrimental to musculoskeletal-injury risk due to walking with load carriage for long periods of time. For example, preliminary experimental studies suggest that exoskeleton use can also affect walking-gait motion (kinematics) and body forces (kinetics), even during short marching periods without additional load (Gregorczyk et al., 2010). Such gait-related changes could lead to unintended musculoskeletal injuries, such as stress fractures, in the lower extremities (van Herpen et al., 2019) or the lower back (Clansey et al., 2012). To date, existing studies that investigated the potential effects of lower-body exoskeleton use with load carriage on walking kinetics and kinematics only focused on their impact on the human body over very short time periods (e.g., less than 10 min, with a maximum walking distance of 1.8 km) (Gregorczyk et al., 2010; Ding et al., 2017). However, load-carriage activities during military operations may last for many hours (Knapik et al., 2012; Department of the Army, 2022). In addition, the exoskeleton devices investigated in these studies provided mechanical power simultaneously at multiple joints, including the hip, ankle, and knee, which prevents us from isolating the effect of the device on a single joint. Studies that were conducted for slightly longer time periods (i.e., up to 30 min, with a maximum walking distance of 2.25 km) and investigated ankle exoskeleton devices either did not involve load carriage (Gordon and Ferris, 2007; Sawicki and Ferris, 2008; 2009a; Malcolm et al., 2013; Mooney et al., 2014b; Koller et al., 2015; Mooney and Herr, 2016; Ingraham et al., 2022; Medrano et al., 2022; Wu et al., 2024) or did not measure or compute gait-related parameters (Mooney et al., 2014a; Zhang et al., 2017; Panizzolo et al., 2019). Thus, previous studies have not considered the important fact that the human body is more susceptible to injury once it is fatigued, such as during the late stages of load carriage (Clansey et al., 2012), preventing us from understanding the true impact of wearing an exoskeleton device for time periods longer than 30 min. Specifically, we do not know to what extent load carriage and walking distance affect exoskeleton-induced alterations, if any, in gait parameters and whether such alterations are detrimental or beneficial to the human body.

Computational modeling complements experimentation and can be used as a tool to characterize the body’s biomechanical responses under various conditions to identify risk factors and assess potential strategies to reduce the risk of musculoskeletal injuries. For example, by developing individualized musculoskeletal models based on experimental data, our U.S. Department of Defense (DoD) team has previously characterized the effects of stature and load carriage on the risk of stress-fracture injury to the tibia in men and women while running or walking (Xu et al., 2016; Xu et al., 2017; Xu et al., 2020; Unnikrishnan et al., 2021; Rubio et al., 2023; Sundaramurthy et al., 2023). In addition, using computational modeling, we have evaluated the impact of dozens of modifiable gait parameters on stress-fracture risk to identify potential mitigation strategies. For instance, for a simulated 10-week U.S. Army Basic Combat Training regimen, we found that reducing stride length by 10% during running decreases the stress-fracture risk to the tibia in healthy, young women by an average of 60% (Sundaramurthy et al., 2023).

To extend the scope of previous assessments of active ankle exoskeleton devices, we designed a pilot study to characterize the effects of a comparatively longer use of an active, wearable ankle exoskeleton (i.e., the ExoBoot EB60; Dephy, Inc., Boxborough, MA) on the walking biomechanics of adult military-age men with load carriage. Compared to previous works, this study was roughly twice as long in distance (i.e., 5 km) and in time duration (i.e., 60 min). Here, we hypothesized that walking with the ExoBoot for 5 km while carrying a 22.7-kg load will have largely beneficial effects on the joint kinematics and kinetics of the lower leg of young, healthy men compared to walking without the device. Towards this end, we developed individualized musculoskeletal models using newly collected experimental data for five men and investigated the changes in the spatiotemporal parameters, kinematics (i.e., the joint angles), and kinetics (i.e., the joint forces and moments) of the lower-extremity joints (i.e., the hip, knee, and ankle) for these participants during walking on a level treadmill for 5 km carrying a 22.7-kg load, with or without wearing the ExoBoot device.

## 2 Materials and methods

### 2.1 Study preparation, image acquisition, and motion-capture data collection

**FIGURE 1:**
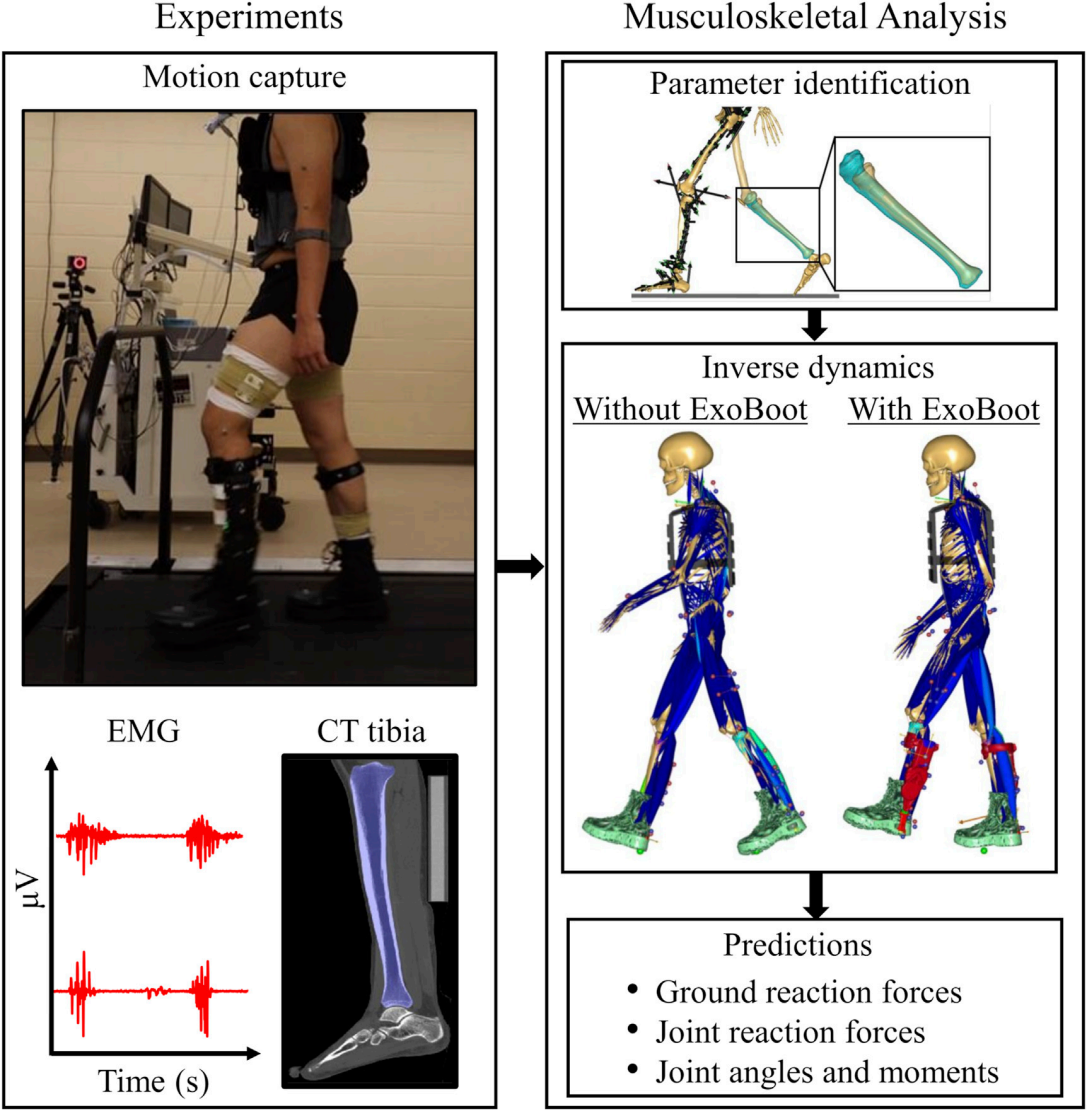
Summary of the integrated experimental and computational study design. We performed a laboratory study on a level treadmill and collected motion-capture (using 52 reflective markers), ground reaction force (GRF), and electromyography (EMG) data from five participants as they walked 5 km with and without an active ankle exoskeleton device (the ExoBoot EB60), while carrying a 22.7-kg load. We collected EMG, GRF, and motion-capture data for 20 s at the baseline (i.e., 0 km) and at every 1-km mark thereafter. We also collected computed tomography (CT) scans of the tibia and lumbar vertebrae L4-L5 from each participant. To develop the computational model, we first incorporated participant-specific anthropometric, tibial, and lumbar spine data into the musculoskeletal model and performed a parameter identification analysis to personalize the model for each participant. Next, we performed kinematic and inverse dynamics simulations for two conditions, i.e., walking with and without the ExoBoot, while carrying the 22.7-kg load. We used the simulation results to compute the GRFs, joint reaction forces, joint angles, and joint moments for each participant under each walking condition.

We enrolled five young, healthy men between the ages of 18 and 25 years, as representative of military recruits in accordance with an anthropometric survey of U.S. Army Soldiers (Department of the Army, 2011; Knapik et al., 2012). All participants reported that they were experienced treadmill walkers and free of injuries that would limit their ability to be physically active for at least 3 months prior to participating in the study. The study protocol was approved by the University of Kentucky Institutional Review Board and by the Office of Human Research Oversight of the U.S. Army Medical Research and Development Command, Fort Detrick, MD. Prior to data collection, we obtained a written informed consent from each participant and recorded their age, mass, height, foot length, body fat percentage, and body mass index (BMI) (Table 1). We also collected quantitative computed tomography (CT) images of each participant’s left tibia and lumbar vertebrae segments L4-L5 using a Siemens CT scanner (Siemens Medical Solutions, Malvern, PA) (Figure 1). The scans had an in-plane pixel resolution of 0.49 × 0.49 mm^2^ and a slice thickness of 0.60 mm. Each CT scan included a calibration phantom of known calcium hydroxyapatite concentration (QRM, Moehrendorf, Germany) in the field of view (Figure 1).

**TABLE 1:** Anthropometric characteristics of 5 young, healthy men.

	Age (years)	Mass (kg)	Height (m)	Foot length (m)	Body fat (%)	BMI (kg/m^2^)
Mean (±SD)	22.0 (1.2)	85.2 (9.5)	1.77 (0.04)	0.27 (0.05)	15.3 (6.5)	27.1 (2.5)
Range	21–24	77.8–101.3	1.71–1.81	0.26–0.27	6.1–23.0	25.2–31.3

The data are presented as means [±1 standard deviation (SD)] or range. BMI: body mass index.

Each participant completed four walking trials in the same order spread across four separate days. During each trial, participants walked at a constant speed of 1.34 m/s on a leveled instrumented treadmill (Bertec Corporation, Columbus, OH) in the following order: *1*) walking without any additional weight and without wearing the ExoBoot, *2*) walking with a 22.7-kg load (50 lb) without the ExoBoot, *3*) walking without any additional weight while wearing the ExoBoot EB60, and *4*) walking with a 22.7-kg load while wearing the ExoBoot EB60. In addition, prior to the first trial with the ExoBoot (i.e., visit *3*), each participant completed a training visit to familiarize themselves with walking while wearing the ExoBoot device. During this visit, we performed alignment and adjustments of the shin pad for each participant. Moreover, to ensure that the participants were acclimated to walking with the ExoBoot on a force-plate instrumented treadmill, we allowed them to walk until they reported being comfortable with the device’s assistance for a minimum of 10 min. We did not collect any data during the training visit.

**FIGURE 2:**
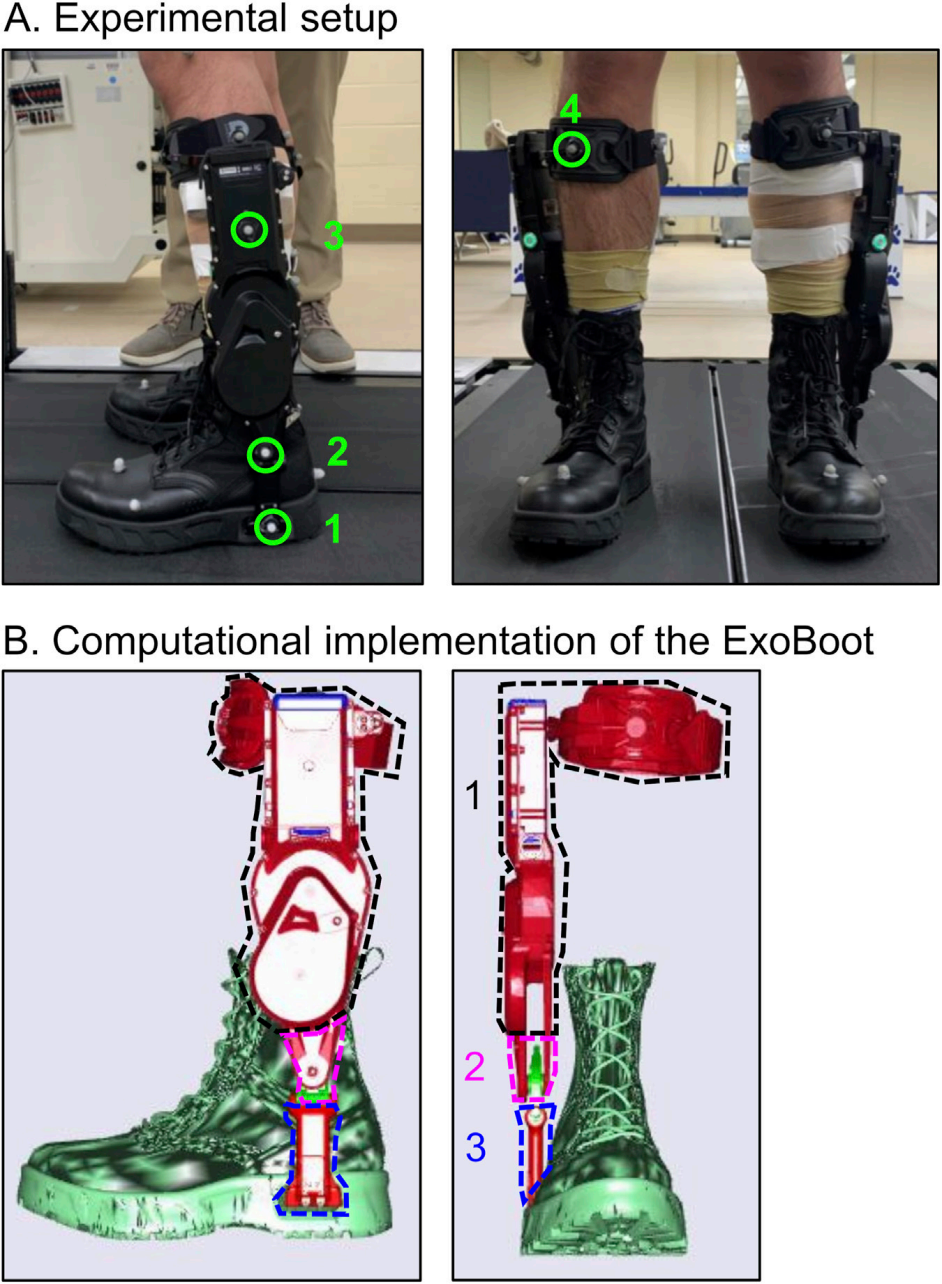
**(A)** Experimental setup describing the locations of the four optical markers placed on the ExoBoot for motion capture during trials in which participants wore the exoskeleton device: *1*) sole where the ExoBoot connected to the boot, *2*) ankle joint, *3*) below the battery indicator, and *4*) anterior part of the shin pad. **(B)** Implementation of the ExoBoot in the musculoskeletal model as three rigid-body segments. Segment 1 (black dashed line), Segment 2 (pink dashed line), and Segment 3 (blue dashed line) represent the upper, middle, and lower frames of the ExoBoot.

We chose to evaluate an active ankle exoskeleton because preliminary assessments of the ability of lower-body exoskeletons to enhance user mobility conducted by the U.S. Army and other groups have shown promising results (Sawicki and Ferris, 2008; Gregorczyk et al., 2010; Mooney et al., 2014a). In addition, we selected the walking speed of 1.34 m/s as representative of the various foot marches conducted in the U.S. Army (Department of the Army, 2022). For the trials with a 22.7-kg load carriage, participants wore a vest strapped to their upper body with the load symmetrically distributed between the front and back because during foot marches military personnel carry approximately symmetrical loads >90% of the time (Figure 1) (Knapik et al., 2012). For trials with and without the ExoBoot device, participants wore slightly modified military boots with custom carbon-composite inserts embedded in the lateral side of the soles. For the trials with the device, participants wore the ExoBoot EB60 on the lateral side of each leg and rigidly secured them to the shins via a shank attachment and a strap secured just below the tuberosity of the tibia and whose distal ends were attached to the carbon-composite inserts in the boots (Figure 2A). The ExoBoot had an inversion-eversion joint and a plantar-dorsiflexion joint and used a built-in brushless electric motor and flat cable transmission to provide plantarflexion assistance during walking. Specifically, each side of the ExoBoot applied a torque to the participant’s ankle by tightening a strap between the motor and the boot during the push-off portion of the gait cycle to provide a burst of positive power during the terminal stance phase. In addition to the training visit to acclimatize themselves to walking with the ExoBoot, before each trial involving ExoBoot use, participants underwent a warm-up period to calibrate the device-applied torque. We included this warm-up period to ensure that the torque assistance provided to each participant was appropriate for the specific trial (e.g., with load vs. without load). The warm-up calibration period typically consisted of walking approximately 20–30 steps at a consistent pace on a flat surface to determine the amount of torque to be applied, which was dependent on the participant’s gait. After the calibration was complete, the participant-specific torque was applied by ramping it up over the first 3–5 steps (the maximum torque was set to 35 N·m).

Following warm up for each trial, participants walked 5 km in ∼60 min, during which we collected motion-capture data, ground reaction forces (GRFs), and electromyography (EMG) data for ∼20 s at baseline (i.e., at 0 km) and at each 1-km mark thereafter (Figure 1). In total, we collected 120 s of data during each walking trial. We collected GRF data from the instrumented treadmill (Bertec Corporation, Columbus, OH) and EMG data (Trigno Avanti, Delsys Inc., Natick, MA) from eight major lower-extremity muscles at 2,000 Hz, including the gluteus medius, biceps femoris, vastus lateralis, rectus femoris, vastus medialis, gastrocnemius, soleus, and tibialis anterior. In addition, we continuously collected data from the ExoBoot, including velocity, acceleration, and torque, measured by the device throughout the whole walking trial. We manually flagged the ExoBoot data at every 1-km mark to guarantee synchronization with the force-plate, motion-capture, and EMG data. The data collection length of 20 s provided a sufficient number of strides (∼15–25) to obtain consistent stance and swing durations for all conditions (Rubio et al., 2023; Tong et al., 2023). We collected motion-capture data from 52 reflective markers (Motion Analysis Corporation, Santa Rosa, CA) at a sampling frequency of 200 Hz. We placed the markers bilaterally on anatomical landmarks and segments throughout the body, including the arms, trunk, pelvis, thighs, shanks, and feet. In addition, we placed three tracking markers on the back of each boot to identify the proximal, distal, and lateral heels. We also placed additional tracking markers on the right anterior thigh and shin as well as on the second head of the metatarsal bone to identify the right vs. the left side of the foot (Figure 2). In the trials where participants wore the exoskeleton device, we placed four markers at the following locations on the ExoBoot: *1*) on the sole where the ExoBoot connected to the boot, *2*) on the ankle joint, *3*) below the battery indicator, and *4*) on the anterior of the shin pad (Figure 2A). We collected the ExoBoot torque data at 200 Hz.

### 2.2 Individualized musculoskeletal models

Similar to our prior studies (Xu et al., 2016; Xu et al., 2017; Xu et al., 2020; Taylor et al., 2021; Unnikrishnan et al., 2021; Rubio et al., 2023; Sundaramurthy et al., 2023), we developed individualized musculoskeletal models using the AnyBody Modeling System (AnyBody Technology, Aalborg, Denmark) to determine the joint kinematics and kinetics for each participant. Briefly, the software provided a generic musculoskeletal model of an average male consisting of rigid segments, including arms, trunk, pelvis, thighs, shanks, and feet, as well as 169 muscles in the lower extremities. To individualize the generic model for each participant, we first morphed the generic tibial and lumbar vertebrae L4-L5 geometry in the AnyBody model to match the participant-specific tibial and lumbar L4-L5 geometry extracted from the CT scan using Mimics (Materialise, Leuven, Belgium). Specifically, we used the automated segmentation in Mimics to segment the bone, followed by a manual refinement to generate a clean tibial and lumbar L4-L5 geometry. Next, we scaled the other segments in the generic musculoskeletal model based on the anthropometric measurements (e.g., mass, height, foot length, and body fat percentage) of each participant. We defined 42 markers in the musculoskeletal model at locations corresponding to where they were placed on the participants during the experimental walking trials.

For trials that involved participants carrying a load or wearing the ExoBoot, we modeled the vest and the ExoBoot as rigid body segments in AnyBody. Specifically, we first modeled the ExoBoot as three rigid body segments (Figure 2B), with a total weight of 12 N, using the material properties (i.e., mass and inertia tensor) provided by the manufacturer. The first segment (Figure 2B, black dashed line) represented the upper frame of the ExoBoot, which we coupled from the center of the knee to the center of the ankle of each leg in the musculoskeletal model. The second and third segments (Figure 2B, pink and blue dashed lines, respectively) represented the lower frames of the ExoBoot, which we coupled from the ankle to the sole of the foot of each leg in the musculoskeletal model. Then, we defined two revolute joints on the ExoBoot, which allowed for ankle rotation around the z-axis (ankle dorsiflexion and plantarflexion) and the x-axis (ankle eversion and inversion) on each foot. Finally, we defined four markers in the musculoskeletal model at locations corresponding to where they were placed on the ExoBoot during the experimental walking trials (Figure 2A).

We applied an optimization scheme that minimized the errors between the markers tracked in the experiment and the markers defined in the model, to further optimize the body segments’ lengths. Once optimized, using the 20-s marker-tracking data collected for each walking trial, we used the participant-specific musculoskeletal model to compute body motion (i.e., the joint angle changes throughout the entire body), including the kinematics of the lower-extremity joints (e.g., hip, knee, and ankle). Finally, we determined the kinetics of the hip, knee, and ankle by performing an inverse dynamics analysis and normalizing the GRFs and joint reaction forces (JRFs) by body weight and the joint moments by body mass. We provided GRFs in the vertical direction relative to the ground because they had by far the highest magnitude.

### 2.3 Assessment of kinematic and kinetic parameters

We used the results of the kinematic and kinetic analyses to determine the impact of two main effects, i.e., walking distance (0 km vs. 5 km) and exoskeleton device use (with ExoBoot vs. without ExoBoot), on the biomechanics of each individual participant. When comparing changes in kinematic and kinetic parameters due to walking distance, both with and without the ExoBoot, we only considered the two trials where participants carried a 22.7-kg load, because the capability to transport heavy loads with ease is one of the potential benefits of exoskeleton devices. For both trials (with and without the ExoBoot), we considered 0 km to be the baseline for comparison purposes. For each participant, using the 20-s GRF data during walking from AnyBody comprising ∼15–20 strides, we first identified the start and end points of each stride (and, hence, the stride duration) at 0 km (i.e., baseline) and at 5 km by using a threshold value of 25 N to identify the peaks in the GRFs. Next, using the start and end points of each stride, we extracted 26 distinct parameters per stride, including spatiotemporal (e.g., stride duration and normalized stride length) as well as joint kinematic and kinetic parameters (e.g., joint angles, moments, and JRFs). Finally, we performed statistical analyses to evaluate changes in these parameters between the different conditions. For these analyses, we resampled the time history of each stride so that strides of different durations (for different participants during different trials) were each represented by 100 values constituting one gait cycle.

### 2.4 Statistical analysis

Before the study, we performed a power analysis using the G*Power software (v3.1.9.4) to estimate the sample size needed to detect changes in the biomechanical parameters during walking with and without the ExoBoot in young, healthy men. We computed the sample size based on a report by Gregorczyk et al. (2010), which described changes in kinematic and kinetic parameters when participants marched while wearing an exoskeleton suit and carrying a load for 8 min. Because they measured these parameters over a short period compared to ∼60 min in our study, we assumed a larger effect size (ES = 1.00) than the median ES of 0.75 calculated based on their work. Assuming a correlation among repeated measurements of 0.5 and a non-sphericity correction of 1.0, we determined that a sample size of five participants was needed to achieve 80% power at a significance level of 5%.

**FIGURE 3:**
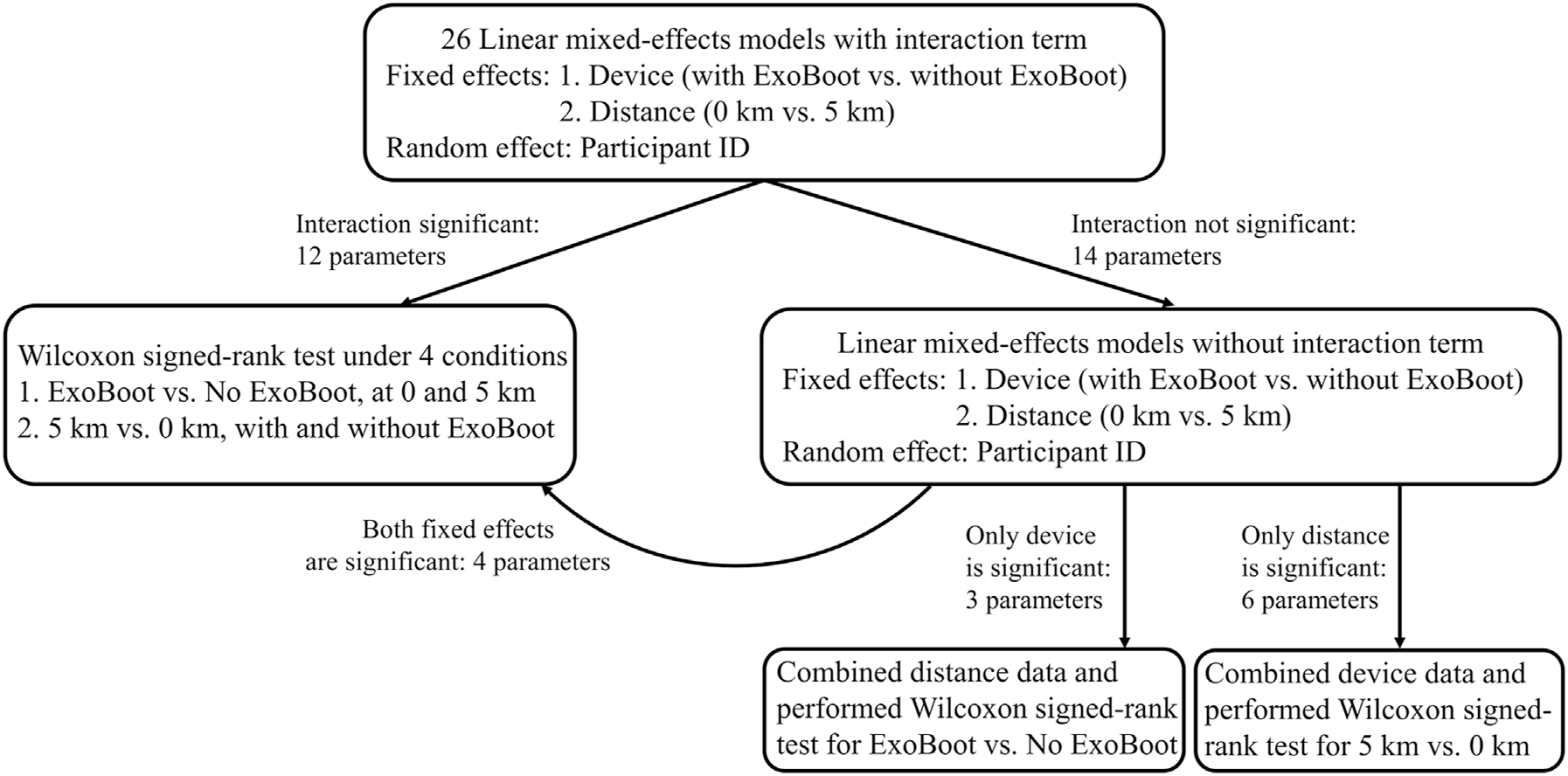
Flowchart of the steps performed to quantitatively assess the impact of wearing the active ankle-powered ExoBoot exoskeleton device on lower-limb biomechanical parameters after walking for 5 km with a 22.7-kg load.

To determine the impact of walking with the ExoBoot on the biomechanical responses, we first developed linear mixed-effects (LME) models while considering each of the 26 biomechanical parameters as the dependent variable. In the models, we included device use (with ExoBoot or without ExoBoot) and distance (0 km or 5 km) as fixed categorical effects (both with and without a device-distance interaction effect) and the participants as a random effect (intercept only). Next, for each biomechanical parameter (listed in the first column in Table 2), we determined the significance of the interaction effect using the likelihood ratio test. Briefly, for each parameter, we compared the corresponding LME model with and without the device-distance interaction effect. When the interaction effect was not statistically significant, we determined the significance of each fixed effect by comparing the LME model with only one fixed variable (i.e., device or distance) against an LME model without any fixed effect (or an empty model) (Figure 3).

**TABLE 2:** Spatiotemporal parameters, peak joint angles, ground reaction force, and joint kinetics.

	Without ExoBoot		With ExoBoot		Interaction		No interaction	
	0 km	5 km	0 km	5 km	LR statistic	*p* value	Distance *p* value	Device *p* value
Normalized stride length								
	1.44 (0.03)	1.49 (0.05)	1.47 (0.06)	1.50 (0.04)	11.48	**<0.001**	**—**	**—**
Stance duration (s)								
	0.67 (0.01)	0.67 (0.01)	0.68 (0.01)	0.68 (0.01)	**—**	**—**	0.542	**<0.001**
Ground reaction force (BW)								
	1.46 (0.08)	1.49 (0.05)	1.53 (0.09)	1.52 (0.06)	**18.22**	**<0.001**	**—**	**—**
Joint angle (degrees)								
Hip								
Add	6.05 (1.43)	6.73 (2.67)	4.91 (1.95)	5.70 (1.55)	**—**	**—**	**<0.001**	0.204
Abd	12.38 (2.47)	13.56 (5.10)	13.20 (2.86)	15.54 (3.77)	9.01	**<0.001**	**—**	**—**
Ext	19.34 (4.97)	21.83 (6.62)	19.98 (4.52)	21.82 (4.59)	**—**	**—**	**<0.001**	**<0.001**
Fle	29.61 (3.08)	29.37 (5.11)	30.66 (4.03)	29.03 (4.24)	1.69	**<0.001**	**—**	**—**
Knee								
Ext	5.60 (2.80)	4.51 (4.19)	3.67 (3.66)	2.97 (3.23)	**—**	**—**	**<0.001**	**<0.001**
Fle	74.83 (1.57)	76.21 (0.96)	75.33 (0.62)	76.13 (1.27)	**—**	**—**	**<0.001**	0.151
Ankle								
DF	12.43 (4.39)	13.55 (3.98)	12.61 (4.39)	13.99 (4.36)	**—**	**—**	**<0.001**	0.080
PF	12.18 (6.88)	12.71 (7.42)	18.16 (4.31)	18.08 (3.84)	**—**	**—**	0.354	**<0.001**
Trunk								
Ext	7.02 (2.95)	10.47 (1.65)	5.70 (1.84)	7.00 (0.96)	24.85	**<0.001**	**—**	**—**
Fle	10.48 (2.85)	14.08 (2.63)	9.23 (2.32)	10.75 (1.14)	26.88	**<0.001**	**—**	**—**
Joint reaction force (BW)								
Hip	4.91 (0.66)	5.13 (0.41)	5.01 (0.62)	5.00 (0.45)	**8.18**	**<0.001**	**—**	**—**
Knee	5.18 (0.67)	5.33 (0.82)	5.38 (0.65)	5.51 (0.73)	**—**	**—**	**0.002**	**<0.001**
Ankle	6.81 (0.56)	7.04 (0.39)	6.91 (0.25)	7.04 (0.23)	**—**	**—**	**<0.001**	0.081
Joint moment (N·m/kg)								
Hip								
Abd	0.81 (0.29)	0.91 (0.21)	0.81 (0.29)	0.83 (0.12)	**6.74**	**0.010**	**—**	**—**
Add	0.40 (0.21)	0.37 (0.17)	0.42 (0.13)	0.34 (0.09)	**—**	**—**	**<0.001**	0.595
Ext	1.22 (0.30)	1.27 (0.19)	1.22 (0.22)	1.23 (0.18)	**4.10**	**0.040**	**—**	**—**
Fle	0.56 (0.17)	0.51 (0.09)	0.63 (0.11)	0.65 (0.13)	**14.87**	**<0.001**	**—**	**—**
Knee								
Ext	0.74 (0.20)	0.83 (0.11)	0.77 (0.24)	0.77 (0.13)	**11.24**	**<0.001**	**—**	—
Fle	0.75 (0.17)	0.80 (0.20)	0.81 (0.15)	0.85 (0.14)	**—**	**—**	**<0.001**	**<0.001**
Ankle								
DF	0.25 (0.05)	0.24 (0.01)	0.26 (0.06)	0.26 (0.06)	—	—	0.613	**0.040**
PF	1.83 (0.12)	1.87 (0.14)	1.84 (0.07)	1.85 (0.14)	**—**	**—**	**0.009**	0.548
ST eve	0.40 (0.12)	0.41 (0.07)	0.42 (0.05)	0.42 (0.07)	**—**	**—**	**—**	—
ST inv	0.09 (0.04)	0.13 (0.03)	0.11 (0.03)	0.12 (0.04)	**20.59**	**<0.001**	**—**	—

The data for the parameters are represented as mean values (1 standard deviation) across the five participants. For each participant, we obtained the parameter value by averaging over 15−20 strides for each condition. Bold *p* indicates a statistically significant main effect (device or distance) or a significant interaction between the main effects based on the likelihood ratio test performed on the linear mixed-effects models. Abd, abduction; Add, adduction; BW, body weight; DF, dorsiflexion; Ext, extension; Fle, flexion; PF, plantarflexion; ST eve, subtalar eversion; ST inv, subtalar inversion.

For each of the five participants, using the 20-s data from AnyBody comprising ∼15–20 strides, we first calculated the mean ± standard deviation (SD) of the peak value for each of the 26 computationally derived biomechanical parameters at 0 and 5 km during each of the two walking trials (with and without the ExoBoot device). For the subset of parameters that achieved significance for both the device and distance effects or for their interaction, we performed four pairwise comparisons of their mean values across the five participants using a non-parametric test, the Wilcoxon signed-rank test (Fagerland, 2012), with a Benjamini-Hochberg correction for multiple comparisons (Benjamini and Hochberg, 1995). We compared the two device conditions (i.e., with ExoBoot vs. without ExoBoot) at 0 km and at 5 km and the two distance conditions (i.e., 5 km vs. 0 km) with and without the ExoBoot (Figure 3). For parameters that achieved significance in only one of the fixed effects (device or distance), we grouped the data for the non-significant effect and performed a Wilcoxon signed-rank test to compare the mean values of the significant effect across the five participants. Given the small sample size (N = 5) of our study and that the derived biomechanical parameters included instances of normally and not-normally distributed parameters, we chose to use a non-parametric, distribution-free test to circumvent the possibility of violating the assumption of normality while still having sufficient power to identify differences in each parameter between two dependent samples.

Finally, we also calculated the ES (i.e., Cohen’s *d*) (Lakens, 2013) to evaluate differences in the means of each of the 26 biomechanical parameters between the two device conditions (i.e., with ExoBoot vs. without ExoBoot) at 0 km and 5 km and between the two distance conditions (i.e., 5 km vs. 0 km) with and without wearing the ExoBoot. We performed the statistical analyses for the LME models using the RStudio v1.4 statistical software, including the lme4, lmerTest, and emmeans packages, with an alpha level of 0.05. We performed the Wilcoxon signed-rank test using the *signrank* function with an alpha level of 0.05 and calculated Cohen’s *d* using the *meanEffectsize* function in MATLAB R2022b (MathWorks, Natick, MA).

## 3 Results

### 3.1 Muscle activities

**FIGURE 4:**
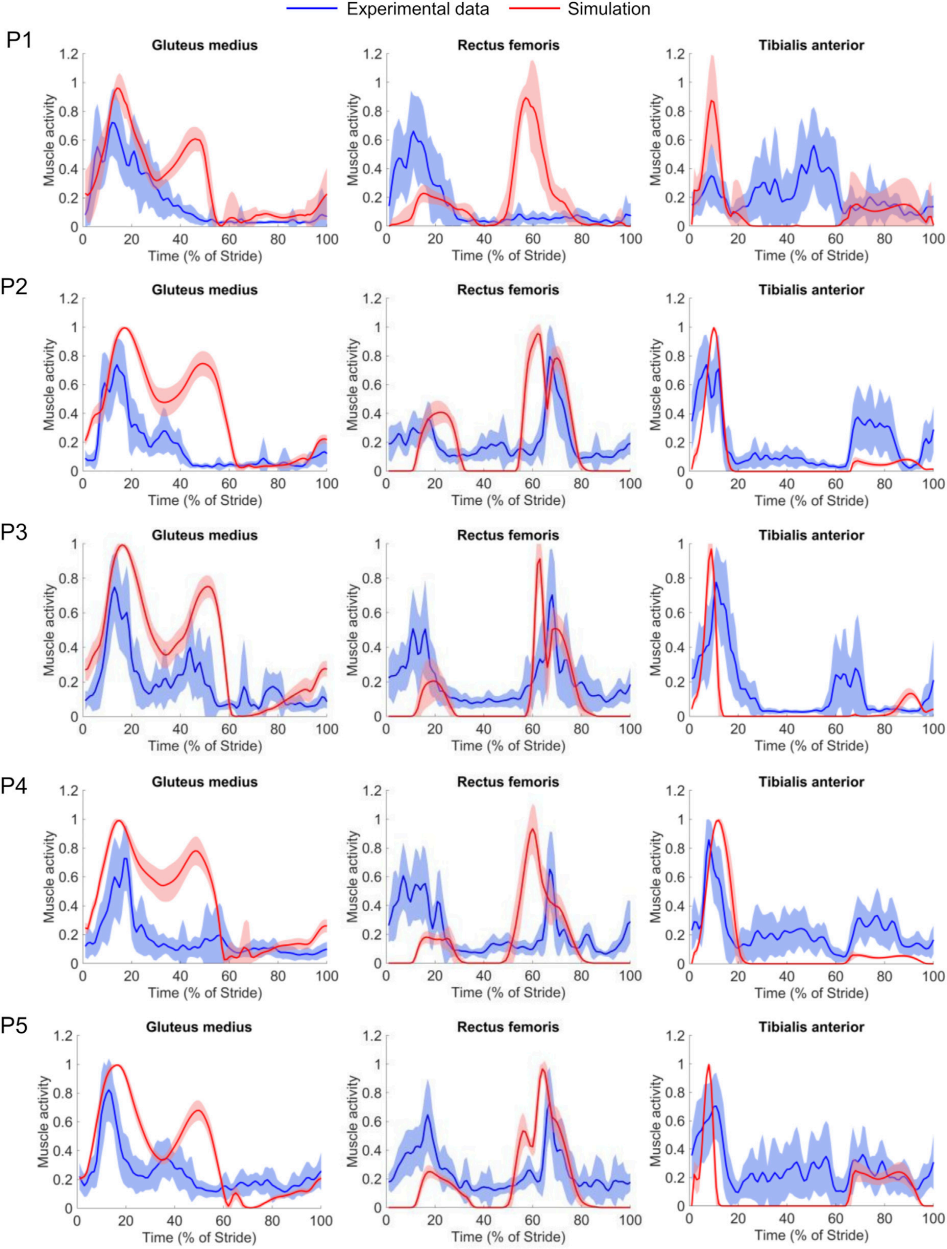
Comparison of muscle activities predicted by the musculoskeletal model (red solid lines) and the electromyography (EMG) data measured in the experiment (blue solid lines) for the gluteus medius, rectus femoris, and tibialis anterior muscles as a function of the percent of the stride for each of the five participants. The shaded areas represent the stride-averaged EMG envelopes (mean ± 1 standard deviation) for each participant (P#). We separately normalized the magnitudes of the muscle activity and the EMG recordings to the maximum of each stride, for each participant.

To assess the validity of the musculoskeletal models, we qualitatively compared the time courses of the predicted muscle activity with the EMG recordings at 0 km when the participants did not carry any load and did not wear the ExoBoot. We did not perform a quantitative comparison because the model-predicted muscle activity and the experimentally measured EMG do not represent the same quantity. Specifically, the EMG provides a measure of muscle electrical activity in response to a nerve’s stimulation, whereas in the computational model we computed muscle activity as the force generated within a muscle for a particular motion divided by its maximum strength. Thus, while the EMG and muscle activity are distinct quantities, they both indicate the level of muscle activation and can be qualitatively compared. Specifically, we chose to perform the comparison for three muscle groups, i.e., tibialis anterior, rectus femoris, and gluteus medius, to ensure we included different muscle groups that are activated during both the swing and stance phases of a gait cycle. For this comparison, we normalized the EMG and muscle activity values in each stride by their maximum values. This allowed us to qualitatively compare when a particular muscle group was most active during a walking stride as measured by the EMG recordings and as predicted by the musculoskeletal models (Figure 4; blue, EMG measurements; red, model predictions).

For certain phases of the stride for participants P2−P5, we observed a reasonable qualitative agreement between the predicted and measured muscle activity profiles after considering the electromechanical delay, which represents the time lag between the onset of muscle activation (measured by EMG) and muscle force generation (predicted by the models) (Cavanagh and Komi, 1979). Specifically, the models correctly predicted activation of the ankle dorsiflexor muscles (tibialis anterior) during the late-swing and early-stance phases. Consistent with the EMG recordings, the models predicted the onset of knee extensor (rectus femoris) activity at mid-stance to absorb shock and the onset of hip abductor (gluteus medius) activity in the early- and mid-stance phases. We did observe some inter-subject variability in the EMG recordings (Figure 4, blue lines). For example, for participant P1, at mid-stance we did not observe activation of the rectus femoris or gluteus medius, both of which were activated in the mid-stance phase for the remaining four participants (P2−P5). In addition, we also observed irregular tibialis anterior activity for this participant during mid-stance, a pattern we did not notice in the EMG recordings of the other four participants.

### 3.2 Spatiotemporal and joint kinematic parameters

The average torque provided by the ExoBoot to the participants’ ankle varied between 18 and 24 N·m. For the normalized stride length, we found that the device-distance interaction effect was statistically significant (*p* < 0.001) (Table 2). However, the mean value of this parameter did not significantly change between the two devices (*p* ≥ 0.438) or between the two distances (*p* ≥ 0.125) (Table 3). In terms of ES, the normalized stride length increased considerably at 5 km compared to baseline when the participants walked without the ExoBoot, with an ES of 1.17 (Table 3) [5 km: 1.49 (0.05) vs. 0 km: 1.44 (0.03) (Table 2)]. In contrast, for stance duration, only the device effect was statistically significant (*p* < 0.001) (Table 2). In fact, compared to without the ExoBoot, walking with the ExoBoot significantly increased the stance duration (*p* < 0.001), with an ES of 1.12 (Table 4). The stance durations at 5 km and 0 km were the same within a device condition (Table 2).

**TABLE 3:** Pairwise comparisons of spatiotemporal, joint kinematic, and joint kinetic parameters with significant interaction between device and walking distance.

	Device effect (ExoBoot vs. No ExoBoot)				Distance effect (5 km vs. 0 km)			
	0 km		5 km		With ExoBoot		Without ExoBoot	
	*p* value	ES	*p* value	ES	*p* value	ES	*p* value	ES
Normalized ground reaction force (BW)								
	0.063	0.75 [−0.45, 1.90]	0.125	0.42 [−0.73, 1.55]	1.000	−0.12 [−1.23, 1.01]	0.188	0.44 [−0.71, 1.56]
Normalized stride length								
	0.438	0.57 [−0.60, 1.70]	0.625	0.17 [−0.96, 1.28]	0.188	0.56 [−0.61, 1.69]	0.125	**1.17 [**−**0.11, 2.39]**
Joint angle (degrees)								
Hip Abd	0.438	0.28 [−0.86, 1.40]	0.313	0.40 [0.75, 1.52]	0.125	0.63 [−055, 1.77]	1.000	0.27 [−0.87, 1.38]
Hip Ext	0.813	0.12 [−1.00, 1.24]	0.813	0.00 [−1.12, 1.12]	0.125	0.36 [−0.78, 1.48]	0.063	0.38 [−0.76, 1.51]
Hip Fle	0.625	0.27 [−0.87, 1.38]	1.000	−0.06 [−1.18, 1.06]	0.438	−0.36 [−1.48, 0.79]	1.000	−0.05 [−1.17, 1.07]
Knee Ext	0.313	−0.53 [−1.67, 0.63]	0.125	−0.37 [−1.49, 0.77]	0.313	−0.18 [−1.30, 0.94]	0.438	−0.28 [−1.40, 0.86]
Trunk Ext	0.313	−0.49 [−1.62, 0.67]	0.063	−**2.32 [**−**3.86,** −**0.70]**	0.188	**0.80 [**−**0.40, 1.97]**	0.063	**1.30 [**−**0.01, 2.55]**
Trunk Fle	0.438	−0.43 [−1.56, 0.72]	0.063	−**1.48 [**−**2.78,** −**0.12]**	0.125	**0.75 [**−**0.45, 1.90]**	0.063	**1.18 [**−**0.10, 2.41]**
Joint reaction force (BW)								
Hip	1.000	0.15 [−0.98, 1.26]	0.438	−0.29 [−1.41, 0.85]	0.625	−0.02 [−1.14, 1.10]	0.625	0.37 [−0.77, 1.49]
Knee	0.625	0.27 [−0.86, 1.39]	0.625	0.21 [−0.92, 1.32]	0.625	0.17 [−0.95, 1.29]	0.625	0.18 [−0.95, 1.30]
Joint moment (N·m/kg)								
Hip Abd	1.000	0.00 [−1.12, 1.12]	0.313	−0.44 [−1.57, 0.71]	1.000	0.05 [−1.07, 1.17]	0.625	0.34 [−0.80, 1.46]
Hip Ext	1.000	−0.02 [−1.14, 1.10]	0.063	−0.21 [−1.33, 0.92]	1.000	0.12 [−1.00, 1.24]	0.313	0.17 [−0.95, 1.29]
Hip Fle	0.313	0.46 [−0.69, 1.59]	0.125	**1.15 [**−**0.13, 2.37]**	0.625	−0.02 [−1.14, 1.10]	1.000	−0.37 [−1.49, 0.78]
Knee Ext	0.625	0.14 [−0.98, 1.26]	0.438	−0.44 [−1.56, 0.71]	1.000	−0.02 [−1.14, 1.10]	0.188	0.49 [−0.67, 1.62]
Knee Fle	0.438	0.31 [−0.82, 1.43]	0.625	0.27 [−0.86, 1.39]	0.313	0.24 [−0.89, 1.36]	0.313	0.20 [−0.93, 1.32]
ST inv	0.625	0.50 [−0.66, 1.63]	0.625	−0.20 [−1.38, 0.87]	0.625	0.24 [−0.89, 1.36]	0.188	**1.05 [**−**0.21, 2.25]**

Statistical significance was determined by performing pairwise comparisons using the non-parametric, Wilcoxon signed-rank test (N =5) for four different conditions, i.e., ExoBoot vs. No ExoBoot, at 0 km and 5 km; 5 km vs. 0 km, with and without the ExoBoot. Bold *p* indicates a statistically significant main effect (device or distance).The effect size was calculated using the mean data (derived from ∼15−20 strides) over the five participants. Data are presented as means [95% confidence interval]. An effect size (ES) ≥0.75 is indicated by bold font. Abd, abduction; Add, adduction; BW, body weight; DF, dorsiflexion; Ext, extension; Fle, flexion; PF, plantarflexion; ST inv, subtalar inversion.

**TABLE 4:** Pairwise comparisons of spatiotemporal, joint kinematic, and joint kinetic parameters without significant interaction between device and walking distance.

	Device effect (ExoBoot vs. No ExoBoot)		Distance effect (5 km vs. 0 km)	
	*p* value	ES	*p* value	ES
Stance duration (s)				
	**<0.001**	**1.12 [0.19, 2.02]**	—	—
Joint angle (degrees)				
Hip Add	—	—	0.700	0.36 [−0.49, 1.20]
Knee Fle	—	—	**<0.001**	**0.94 [ 0.04, 1.82]**
Ankle DF	—	—	0.050	0.30 [−0.55, 1.14]
Ankle PF	**<0.001**	**0.99 [0.08, 1.88]**	—	—
Joint reaction force (BW)				
Ankle	—	—	0.060	0.47 [−0.39, 1.32]
Joint moment (N·m/kg)				
Hip Add	—	—	0.230	−0.35 [−1.19, 0.50]
Ankle DF	0.560	0.23 [−0.61, 2.02]	—	—
Ankle PF	—	—	0.380	0.20 [−0.64, 1.04]

Statistical significance was determined by performing pairwise comparisons using the non-parametric, Wilcoxon signed-rank test (N =5) for two different conditions, i.e., ExoBoot vs. No ExoBoot and 5 km vs. 0 km. Bold *p* indicates a statistically significant main effect (device or distance).The effect size was calculated using the mean data (derived from ∼15−20 strides) over the five participants. Data are presented as means [95% confidence interval]. An effect size (ES) ≥0.75 is indicated by bold font. Add, adduction; BW, body weight; DF, dorsiflexion; Fle, flexion; PF, plantarflexion.

Among the joint angles, we found that the device-distance interaction effect was statistically significant (*p* < 0.001) for the peak hip abduction, hip flexion, trunk extension, and trunk flexion angles (Table 2). However, the mean value of each of these four parameters did not significantly change between the two devices or the two distances (Table 3). In terms of ES, compared to baseline, both the peak trunk extension and flexion angles increased at 5 km, with ES values ≥ 0.75, regardless of the ExoBoot condition (Table 3), where the magnitude of the increase in each of these two parameters was smaller when participants walked with the ExoBoot (∼20%) compared to without it (∼42%) (Table 2). In fact, compared to walking without the ExoBoot, walking with it for 5 km considerably reduced the peak trunk extension angle, with an ES of −2.32 (confidence interval = [−3.86, −0.70]), and the peak trunk flexion angle, with an ES of −1.48 (confidence interval = [−2.78, −0.12]), which did not occur at baseline (Table 3).

For the peak ankle plantarflexion angle, we found that only the device effect was statistically significant (*p* < 0.001) (Table 2). Compared to without the ExoBoot, walking with it significantly increased the ankle plantarflexion angle (*p* < 0.001) with an ES of 0.99 (Table 4). For the peak knee flexion angle, only the distance effect was statistically significant (Table 2). Indeed, compared to baseline, walking 5 km significantly increased (*p* < 0.001) this angle with an ES of 0.94 (Table 4). For one of the 26 parameters, the subtalar eversion moment, neither of the two main effects was significant, therefore, we did not perform any pairwise comparisons for this parameter. In summary, while use of the ExoBoot device significantly impacted stance duration and ankle plantarflexion angle regardless of distance, its impact on the peak trunk extension and trunk flexion angles was dependent on the walking distance.

### 3.3 Ground reaction force and joint kinetic parameters

For the GRF and hip JRF, we found that the device-distance interaction effect was statistically significant (*p* < 0.001) (Table 2). With ExoBoot use, the GRF slightly decreased at 5 km compared to baseline, however, this decrease was not significant (*p* = 1.000) (Table 3). For the knee JRF, both the device and distance effects were significant, while the interaction effect was not (Table 2). The JRFs at the hip and knee did not change significantly with ExoBoot use or walking distance (Table 3). For the ankle JRF, only the distance effect was significant. While its mean value increased at 5 km compared to baseline with a moderate ES of 0.47, this change was not significant (Table 4).

For five of the 10 joint moments, i.e., the hip abduction, hip extension, hip flexion, knee extension, and subtalar inversion moments, we found that the device-distance interaction effect was significant (*p* ≤ 0.040) (Table 2). However, none of the mean values of these five parameters changed significantly between the two devices or the two distances (Table 3). In terms of ES, compared to without the ExoBoot, its use increased the mean hip flexion moment by 27% at 5 km with a large ES of 1.15 (Table 3), which was not observed at 0 km. Furthermore, while the hip abduction and hip extension moments consistently increased at 5 km compared to baseline for both device conditions, the magnitude of the increase was considerably smaller when participants wore the ExoBoot (∼2.5% for hip abduction and <1% for hip extension) compared to without it (∼12% for hip abduction and ∼4% for hip extension) (Table 2). When participants walked without the ExoBoot, the subtalar inversion moment increased by 44% at 5 km (Table 2) with an ES of 1.05 (Table 3).

For the peak knee flexion moment, both the device and distance effects were significant (Table 2). However, the pairwise comparison did not show a significant change in the mean values of this parameter between the two devices or the two distances (Table 3). For the ankle dorsiflexion moment, only the device effect was significant (Table 2). Compared to without the ExoBoot, its use increased the mean ankle dorsiflexion moment with a small ES of 0.23, and this increase was not significant (*p* = 0.560, Table 4). For the hip adduction and ankle plantarflexion moments, only the distance effect was statistically significant (Table 2). We found that the mean values of the hip adduction moment decreased (ES: −0.35) and the ankle plantarflexion moment increased (ES: 0.20) at 5 km compared to 0 km, however, neither of these changes was significant (Table 4). In summary, exoskeleton device use considerably increased the hip flexion moment, moderately increased the ankle dorsiflexion moment, and reduced the magnitude of the increase in hip abduction and hip extension moments after a 5-km walk.

## 4 Discussion

The objective of this pilot study was to characterize the biomechanical responses of the lower extremities of young, healthy men after walking with an active ankle exoskeleton for 5 km. Towards this goal, we collected experimental data for five adult men, developed individualized musculoskeletal models, and computed 26 distinct biomechanical responses, including spatiotemporal, joint kinematic, and joint kinetic parameters, for each participant at two distances (0 km and 5 km) as they walked while carrying a 22.7-kg load with and without wearing an active ankle exoskeleton (the Dephy ExoBoot EB60). We found that, irrespective of the distance walked, ExoBoot use significantly increased the stance duration and peak ankle plantarflexion angle (*p* < 0.001; Table 4). In addition, at the 5-km distance, we observed considerable decreases in the peak trunk extension (ES: −2.32) and trunk flexion angles (ES:−1.48) with ExoBoot use compared to without it (Table 3). In general, compared to walking without the ExoBoot, its use reduced the magnitude of the increases in peak trunk extension and trunk flexion angles after walking for 5 km compared to baseline. Finally, irrespective of ExoBoot use, we found that the knee flexion angle significantly increased at 5 km compared to baseline (*p* < 0.001; Table 4).

As expected, we observed large inter-subject variability in certain biomechanical responses of the five participants. For example, at the 5-km distance, compared to without the ExoBoot, its use increased the ankle JRF in two of the participants and decreased it in the other three. Except for a few joint angles, e.g., the peak ankle plantarflexion, trunk extension, and trunk flexion angles, we did not find many parameters that demonstrated the same trend in all five participants when they walked with the ExoBoot. This is consistent with previous studies that have shown an increase in inter-subject variability in gait parameters as individuals become fatigued (Winter et al., 2017; Zandbergen et al., 2023) or when individuals walk with exoskeleton devices compared to unassisted walking (Hybart and Ferris, 2023). Despite the inter-subject variability, we found two parameters that, on average, significantly increased (*p* < 0.001) with ExoBoot use compared to without it. Consistent with previous studies involving both men and women (Sawicki and Ferris, 2009b; Koller et al., 2015), we observed that in comparison with walking unassisted, walking with the ExoBoot significantly increased the peak ankle plantarflexion angle (*p* < 0.001; Table 4). In addition, ExoBoot use significantly increased the average stance duration (*p* < 0.001; Table 4). However, despite being statistically significant, the magnitude of the increase in stance duration was small [with ExoBoot: 0.68 (0.01) s vs. without ExoBoot: 0.67 (0.01) s (Table 2)]. We did not find any previous reports of changes in stance duration due to the use of an exoskeleton device. However, stance duration has been previously shown to decrease in both young and old adults when they walked after becoming fatigued (Zhang et al., 2014; Santos et al., 2019; Halder et al., 2021). Therefore, by increasing the stance duration, exoskeleton use might help mitigate the effect of fatigue.

While previous studies that investigated the impact of active ankle exoskeletons have demonstrated their ability to reduce the energy requirement of walking by ∼8–15%, none of these studies reported any significant changes in the knee or hip joint angles (Sawicki and Ferris, 2008; Malcolm et al., 2013; Mooney et al., 2014b; Koller et al., 2015; Mooney and Herr, 2016; Acosta-Sojo and Stirling, 2022; Gonzalez et al., 2022; Ingraham et al., 2022; Medrano et al., 2022; Wu et al., 2024). In contrast, our results showed that compared to baseline, walking 5 km significantly increased the knee flexion angle regardless of ExoBoot use (*p* < 0.001; Table 4). In addition, while not statistically significant, our results showed that ExoBoot use considerably altered certain hip and knee joint angles at 5 km, which did not occur when participants walked unassisted. In particular, compared to baseline, the hip flexion angle decreased (ES: −0.36) and the hip abduction angle increased (ES: 0.63) after participants walked 5 km with the ExoBoot (Table 3). It is possible that because of the longer walking time and distance that the participants walked with an active exoskeleton in our study [∼60 min (i.e., 5 km)] compared to previous studies [up to ∼30 min or ∼2 km], we were able to capture changes in hip joint angles that were not previously reported.

On the other hand, some of our findings regarding the effect of the exoskeleton device on joint kinetics are corroborated by previous studies. For example, Mooney and Herr (2016) reported that, compared to unassisted walking, walking with an active ankle exoskeleton for ∼2 km in ∼20 min increased the peak knee flexion and the peak hip flexion moments during late stance and decreased the peak hip extension moment in healthy men, which is consistent with our results (Table 3). In another study, compared to unassisted walking for ∼2 km in ∼30 min, walking with an active ankle exoskeleton increased the average positive hip power (obtained by multiplying the hip angular velocity by the hip torque) in healthy men, implying that an exoskeleton could potentially reduce the effort in the hip muscles during walking (Koller et al., 2015). However, in these studies, participants did not carry a load while walking. Consistent with these two studies, we found that, compared to unassisted walking, on average, wearing the ExoBoot for 5 km also increased the peak hip flexion moment (ES: 1.15) and lowered other moments around the hip joint [i.e., the hip abduction (ES: −0.44) and the hip extension (ES:−0.21) (Table 3)].

In our analysis, we also found eight parameters that, on average, demonstrated the same trend (i.e., an increase or a decrease) at 5 km compared to 0 km both with and without the ExoBoot. We speculate that these increases could be due to fatigue in some, if not all, of the participants because they occurred irrespective of ExoBoot use and because the distance walked in our experiments (i.e., 5 km) was longer than that considered as a mark for fatigue onset (i.e., 3 km) in previous fatigue-inducing running protocols that did not involve load carriage (Winter et al., 2017). Among the spatiotemporal and joint angle parameters, we found that the peak knee flexion angle increased significantly at 5 km compared to 0 km irrespective of ExoBoot use. Indeed, this angle has been previously shown to increase in young, healthy adults during running following fatigue-inducing protocols (Kellis et al., 2011; Bazuelo-Ruiz et al., 2018). Another such parameter, the peak trunk flexion angle, has been previously shown to increase after fatigue onset in running and isokinetic muscle contraction protocols (Granacher et al., 2010; Heiderscheit et al., 2011; Warrener et al., 2021). Interestingly, in our study, although the trunk flexion angle increased both with and without the ExoBoot after 5 km of walking (likely due to fatigue), the magnitude of the increase was smaller when the participants walked with the ExoBoot (16% with ExoBoot vs. 35% without ExoBoot, Table 2). Similarly, the stride length also increased by a smaller magnitude when participants walked for 5 km with the ExoBoot compared to without it (2% with ExoBoot vs. 3.5% without ExoBoot, Table 2). We do know from previous studies that a reduction in stride length when running without load carriage, even for short intervals, is likely to reduce the risk of a stress-fracture injury in healthy, young men and women (Edwards et al., 2009; Sundaramurthy et al., 2023). Therefore, in accordance with our hypothesis, ExoBoot use could be potentially beneficial because a shorter stride length results in a smaller loading to the muscles and bones.

We observed the same trend, i.e., a reduction in the magnitude of the increase with ExoBoot use, in certain hip, knee, and ankle moments: hip abduction (12% without ExoBoot vs. 2% with ExoBoot), hip extension (4% without ExoBoot vs. <1% with ExoBoot), knee extension (12% without ExoBoot vs. <1% with ExoBoot), and subtalar inversion (13% without ExoBoot vs. 9% with ExoBoot). Changes in hip kinetics and kinematics due to fatigue could increase the risk of injury to the lower-leg muscles and joints, especially the knee (Krebs et al., 1998; Williams et al., 2001; Baldon et al., 2009; Ferber et al., 2009; Sangeux, 2019). For example, previous studies have shown that walking after fatiguing the hip abductor muscles increases the knee extension angles and the knee adduction moments in both men and women (Patrek et al., 2011; Tang et al., 2022), and a higher external knee adduction moment has often been observed in patients with pathological knee conditions, such as patellofemoral pain syndrome and knee osteoarthritis (Bolgla et al., 2008; O'Connell et al., 2016). In addition to pathological knee conditions, changes in hip-joint kinematics also contribute to hip muscle strength and the risk of hip-joint injury. For example, an increase in peak hip flexion angle has been associated with a decrease in hip flexor muscle strength (i.e., fatigue) during high-intensity running (Riazati et al., 2020). Thus, with results showing that ExoBoot use, on average, altered the hip joint kinematics and kinetics in a manner potentially associated with lowering injury risk (i.e., decreasing the peak hip flexion angle and increasing the hip abduction angle, as well as lowering the hip abduction and hip extension moments), our study suggests that wearing the ExoBoot could attenuate the negative effects of walking long distances with load carriage, which supports our hypothesis.

Our study has several limitations. First, due to inter-subject variability in the biomechanical responses and the small sample size of this pilot study (N = 5 men), the generalizability of the observed group-level effects is limited. We constrained the study to men because they represent 82% of U.S. Service members (Department of Defense, 2023) and, more importantly, to eliminate sex differences as another source of variability. It is well established that gait biomechanics differ between men and women when walking or running unassisted with load carriage (Bode et al., 2021; Rubio et al., 2023), and we expect such variability to persist with the use of the ExoBoot. Second, we assumed that walking 5 km at 1.34 m/s (i.e., for ∼60 min) is sufficient to induce fatigue, which may not be true for all participants. However, we based our study design on a meta-analysis that included 25 different studies involving both male and female participants where fatigue was assumed to be induced by running without load carriage for at least 3 km or for 30 min (Winter et al., 2017). Third, we conducted the experiments in a controlled laboratory environment using a level treadmill. While this laboratory setup is not fully representative of U.S. Army marching (Department of the Army, 2022), we implemented it to minimize confounding factors and systematically delineate the impact of the ExoBoot on the biomechanical parameters in a controlled environment. Hence, the reported results may differ from those obtained on an uneven terrain, as various studies have reported differences in lower-extremity joint mechanics between unassisted walking on a flat terrain versus an uneven terrain, such as larger flex joint angles and smaller joint extension moments (Lange et al., 1996; Leroux et al., 2002; Lay et al., 2006; McIntosh et al., 2006; Silder et al., 2012; Haggerty et al., 2014). Use of the ExoBoot on an uneven terrain should result in similar differences in hip, knee, and ankle joint angles and moments, which need to be further investigated in a new study.

Fourth, the study participants did not have a history of exoskeleton use, which could have influenced their biomechanics when walking with it for the first time. However, we still consider the participants to be a representative sample of military recruits based on the possibility that new recruits may not have prior experience using active exoskeleton devices that enhance mobility. Furthermore, to minimize the effect of no previous exoskeleton use, participants completed a separate training visit to become acclimated to walking with the ExoBoot. Fifth, we modeled the knee and ankle joints as revolute joints, which only captures their movement in the sagittal plane, because the primary movement of knee and ankle joints occurs in this plane. A study by Marra et al. (2015), which evaluated the performance of revolute and higher-fidelity joints for their ability to represent knee joint biomechanics using musculoskeletal models, found their performance to be comparable. Therefore, we believe that modeling the three-dimensional motion of the knee and ankle joints would not have changed our conclusions. Finally, even though we did not customize muscle strength or explicitly account for muscular fatigue, we individualized the tibial and lumbar (L4-L5) geometry and used each participant’s material properties to individualize the musculoskeletal model. We adjusted the muscle strength based on the height, weight, and fat percentage of each participant. We acknowledge that incorporating participant- and task-specific muscle strengths into musculoskeletal models adds an important dimension of personalization, especially when considering such cases as strenuous military training.

## 5 Conclusion

We quantitatively assessed the potential effects of an active ankle exoskeleton device on the lower-limb biomechanics of young, healthy men due to walking with load carriage for 5 km (∼60 min). We found that, compared to without an active exoskeleton device, walking with the ExoBoot while carrying a 22.7-kg load significantly increased the stance duration and peak ankle plantarflexion angle. In addition, walking with the ExoBoot lowered the magnitude of the increases in the trunk flexion angle and stride length compared to unassisted walking. Despite the small sample size of this pilot study, our results suggest that walking with the ExoBoot may mitigate the effects of fatigue-induced changes in certain biomechanical parameters and alter the hip joint angles and moments in a potentially beneficial manner during extended use. By identifying specific gait-related parameters (i.e., stance duration, trunk and knee angles, and hip moments) that are susceptible to change with exoskeleton use, our study offers potential insights into the biomechanical parameters that should be further evaluated for association with lower-leg musculoskeletal injuries due to use of such augmentation devices. In addition, the knowledge gained in this pilot study could provide a benchmark for future studies and inform the development of standards for safe and effective use of emerging exoskeleton technologies.

## Data Availability

The raw data supporting the conclusions of this article will be made available by the authors, without undue reservation.
